# Transcatheter Aortic Valve Replacement versus Surgical Aortic Valve Replacement: A Review of Aortic Stenosis Management

**DOI:** 10.7759/cureus.6431

**Published:** 2019-12-20

**Authors:** Yasar Sattar, Hiba Rauf, Syeda Beenish Bareeqa, Waqas Ullah, Madhura Myla

**Affiliations:** 1 Internal Medicine, Icahn School of Medicine at Mount Sinai, New York, USA; 2 Internal Medicine, Dow Medical College, Karachi, PAK; 3 Internal Medicine, Jinnah Medical and Dental College, Karachi, PAK; 4 Internal Medicine, Abington Hospital - Jefferson Health, Abington, USA; 5 Cardiovascular Disease, University of New Mexico Health Sciences Center, Albuquerque, USA

**Keywords:** severe aortic stenosis, transcatheter aortic valve implantation, transcatheter aortic valve replacement (tavr), surgical aortic valve replacement

## Abstract

Severe aortic stenosis (AS) affects 3.4% of the elderly over 60 years of age. It presents with exertional dyspnea, syncope, angina, and progression to irreversible congestive heart failure. Early intervention produces a better outcome in preventing the clinical deterioration of AS. The choice of intervention is transcatheter aortic valve implantation or surgical aortic valve replacement (SAVR). The decision should be made after evaluating an individual case based on its clinical features and the user’s experience with transcatheter aortic valve replacement (TAVR). We reviewed available data to illustrate the types of ASs, the background of interventions, current guidelines for TAVR, and its comparison with SAVR in terms of adverse effects.

## Introduction and background

Aortic stenosis (AS) is defined as left ventricular outflow tract obstruction due to the narrowing of the orifice of the aortic valve with a forward velocity of blood at least 2 m/sec. In Europe and the United States, calcific AS holds the most share in causing AS in people older than the sixth decade of life, with the congenital bicuspid valve being the most commonly affected among those in their fourth to sixth decade of experience [1]. The pooled estimated prevalence of AS among the elderly is 12.4% and that of severe AS is 3.4% [2]. Criteria establish severe AS when transvalvular aortic velocity is >4 m/sec when the aortic jet velocity is ≥ 5 m/sec, and the aortic orifice is ≤1 cm2. Severe AS is a significant area of concern due to the poor prognosis and deterioration of inotropy, thus requiring early intervention. AS can initially present with exertional dizziness, dyspnea, reduced exercise tolerance, and progression to irreversible congestive heart failure, syncope, and angina in severe AS. Therefore, early intervention in severe AS has better clinical outcomes. The choice of the optimal mode of response has been a topic of debate for the past decade, with several trials assessing the efficacy and safety of transcatheter aortic valve implantation/replacement (TAVI/TAVR) versus surgical aortic valve replacement (SAVR). Approximately 67,500 surgeries are performed annually for aortic valve replacement (SAVR) [3]. A recent study performed by Osnabrugge et al. suggests an estimated prevalence of 3.4% of severe AS in the elderly population in their seventh decade of life. Out of this, approximately 290,000 people over 70 years of age are potentially high risk for surgery that could be managed by TAVR in Europe and North America, with an annual number of 27,000 new candidates for TAVR. This review highlights AS criteria, the background of procedures, current guidelines for TAVR, comparison of different approaches, and future perspectives in the treatment of AS [2,4].

## Review

Literature search strategy

Relevant medical literature was searched using MEDLINE, Scopus, Ovid SP, and Google scholar. Studies were included based on the availability of full text, literature in the English language, and date published between 2005 and 2019. Studies with inconclusive scientific evidence, literature in a language other than English, and those with unavailable full texts were excluded from this review. The search strategy was based on the combinations of medical subject heading, including “s”, “Transcatheter Aortic Valve Implantation”, “Transcatheter Aortic Valve Replacement”, and “Surgical aortic valve replacement”.

Aortic stenosis

The standard aortic orifice is 3.0 to 4.0 cm2 [5]. AS is specified as the narrowing of the valve orifice and a maximal transvalvular velocity of ≥2 m/sec [1]. The transvalvular pressure gradient through the orifice of the valve reduces as a result of stenosis. As the pressure gradient also depends on transvalvular flow, it is low in a decreased cardiac output state. AS is categorized into three types, mild, moderate, and severe, depending on valve orifice size, transvalvular velocity, and pressure. Mild AS has a valve orifice of >1.5 cm2, a transvalvular pressure of <20 mm Hg, and a flow velocity of 2.5 to 3 m/sec [6]. Moderate AS has a valve orifice of 1.0 to 1.5 cm2 with a transvalvular pressure of 25 to 40 mmHg when the transvalvular flow is at baseline [4]. Severe AS has a valve orifice of <1 cm2, a transvalvular pressure of >40 mmHg, and a flow velocity of > 4 m/sec [3]. The criteria for severe AS have high sensitivity due to a broad basis for inclusion, and therefore many patients who fall in this category are asymptomatic or have mild symptoms. These patients require early intervention as they are at an increased risk of sudden death and have a maximum survival of two to three years [7].

History

Over the decades, SAVR with a mechanical or bioprosthetic valve has been the cornerstone for the management of severe cases of AS to improve heart function and survival [4,8]. In 1980, H.R. Anderson tested a balloon-expandable valve in animals followed by Alain Cribier, who proposed the transfemoral arterial approach in humans in 2000. Since then, many renowned scientists have worked on different approaches until Edwards Lifesciences identified a flexible catheter that passed across the aortic arch through the retrograde transfemoral approach. With continuing advances, Medtronic (Minneapolis, MN) introduced a nitinol-based self-expandable valve called CoreValve. The effectiveness of the Edwards SAPIEN (Edwards Lifesciences, Irvine, CA) and Medtronic CoreValve was proven by implantation of these valves at the aortic valve using the transcatheter approach in the PARTNER (Placement of Aortic Transcatheter Valves) trial and the United States CoreValve Pivotal Trial, respectively. At present, both valves are approved by the U.S. Food and Drug Administration (FDA) and employed in treatment by different institutes [9].

Medical therapy

Medical therapy for AS has not shown any promising results in establishing the efficacy of statins, antihypertensive drugs, and drugs that target phosphate and calcium for AS. However, future research may reveal minimized left ventricle (LV) remodeling in AS through angiotensin-converting enzyme inhibitors, angiotensin-receptor blockers, and sacubitril as post-aortic valve replacement medical therapy [4,8,10].

Current indications for intervention in aortic stenosis

Patients with AS can be categorized as symptomatic or asymptomatic. The decision and choice of intervention depend on symptom severity and associated comorbidities. According to the 2017 European Society of Cardiology guidelines, intervention is indicated in all symptomatic patients with severe high gradient AS. Intervention should also be performed in symptomatic patients with severe low-flow, low-gradient AS with reduced ejection fraction. However, when the intervention does not seem to provide any improvement in the quality of life or survival due to debilitating comorbidities, balloon dilation can be considered [11]. All asymptomatic patients who have severe AS with abnormal exercise tolerance tests or reduced ejection fraction should also undergo intervention [4]. The intervention should be performed in a facility that has a cardiology department, cardiac surgery department, and a heart team consisting of an interventional cardiologist, imaging specialists, cardiothoracic surgeons, cardiac anesthetists, and nurses [12].

Symptomatic patients

The decision between SAVR and TAVI should be based on individual patient preference and weighing of the risk-benefit ratio. Before deciding on the type of procedure, the surgical risk score should be determined using the Society of Thoracic Surgery (STS) score or EuroSCORE (European System for Cardiac Operative Risk Evaluation) [9,12]. These scores give the percentage of predictable poor outcomes based on a patient’s clinical features. If the patient is deemed to be at a low surgical risk (the STS score or EuroSCORE II is <4%), SAVR can be safely performed in symptomatic AS patients. However, if the STS score or EuroSCORE II is >4%, then the multidisciplinary heart team should account for individual patient features. In these cases, TAVI can be considered as a suitable intervention. Another procedure, balloon aortic valvotomy, is a diagnostic and therapeutic intervention. It can be performed as bridge therapy in hemodynamically unstable patients or severe AS patients who urgently need major noncardiac surgery. Later, the patient can undergo SAVR or TAVI when stabilized [4].

Asymptomatic patients

Firstly, the patient should be assessed for symptoms such as exertional dyspnea, chest pain, and dizziness. If the patient has equivocal symptoms, then an exercise stress test should be employed [13]. As TAVI is not recommended in asymptomatic patients with severe AS, SAVR can be performed as an elective procedure in those who are prone to become symptomatic if left untreated [4,10]. The rate of event-free survival at two years is only 30% to 50%; therefore, the importance of serial follow-up to monitor progression should be emphasized [14]. The follow-up investigations can include performance on the exercise stress test, changes in echocardiographic findings, and measurement of brain natriuretic peptide [4]. In a multicentric trial, mortality was assessed in asymptomatic severe stenotic patients who either received early surgery or conservative treatment. The study showed significantly lower mortality rates from cardiovascular causes in the early SAVR group [15].

SAVR procedure

SAVR is an extensive heart surgery performed under general anesthesia on a cardiopulmonary bypass. A median incision is made on the sternum to expose the mediastinum. It is followed by dissecting the aorta and removing the stenotic valves. Finally, the native valves are replaced with either mechanical or bioprosthetic valves [16]. After the surgery, the patient is generally kept in the intensive care unit (ICU) for 24 to 48 hours and for a week in the inpatient unit to monitor for complications.

TAVI pre-procedural evaluation

Transthoracic echocardiography is used to assess the degree of AS, valve calcification, the severity of left ventricular dysfunction, and accompanying valvular abnormalities [4]. If this modality is not sufficient to make the diagnosis, transesophageal echocardiography is used to define the valvular anatomy and caliber further [12]. Nowadays, a computerized tomography (CT) scan is preferred as the diagnostic modality due to its accuracy and precision. The diagnostic evaluation can help the heart team to decide on the dimensions of the prosthetic valve, mode, and route of the intervention [17].

Implantation approaches

TAVI can be performed through several routes, most importantly, transfemoral, transaortic, and transapical. The transfemoral route is considered the default approach with better results than the transapical route [18]. Other routes that can be used in cases of difficult femoral access are transcarotid, transaxillary, and transvenous routes [19].

Anesthesia

TAVR can be performed with the patient under local anesthesia with moderate sedation or awake, invariable general anesthesia, and complete general anesthesia for transfemoral, transapical, and transaortic approaches, respectively [20]. The awake TAVR is considered monitored anesthesia care (MAC) and involves variable sedation, analgesia, and anxiolytics as needed. The choice of anesthesia depends on patient-related factors and area-specific practices. The clinical outcomes are similar between the two approaches (general anesthesia and MAC); however, the MAC approach is associated with shorter hospital stays [21].

Percutaneous access

Previously, TAVI was performed with surgical cutdown at the access site, but with advances in medical technology, the percutaneous approach is used with delivery sheaths as small as 14-16 French [9]. The puncture site is visualized by ultrasound or fluoroscopic angiography [22]. Finally, percutaneous closure should be performed as it is minimally invasive and provides for a shorter hospital stay as compared with suture-based closure [23]. The devices available for percutaneous closure are Prostar (Abbott, Abbott Park, IL), ProGlide (Abbott), and MANTA (Teleflex, Morrisville, NC) [24].

Valve implantation

Initially, balloon valvuloplasty was used as a means of pre-dilatation before valve implantation but has been found to increase the risk of cerebral embolization and severe acute aortic regurgitation [25]. Therefore, it is currently performed in only complex cases of severely calcified aortic valves. As we have improved delivery systems and prosthesis, balloon valvuloplasty can safely be avoided to reduce procedure time and complications [26]. After puncturing the site, a long catheter is inserted over the sheath followed by the aortography to guide valve placement. A Safari or Amplatz Super Stiff guidewire (Boston Scientific, Marlborough, MA) is introduced into the LV, which assists in the deployment of an Edwards SAPIEN or Medtronic valve at the site of the aortic valve. The temporary pacemaker is attached with a Safari wire to test for rapid pacing, during which the valve expands and secures along the native aortic valve annulus. The temporary pacemaker is placed in the right ventricle only if the patient develops atrioventricular block or prolonged QRS duration after the procedure [27].

Post-TAVI management

Patients are monitored for hemodynamic stability and cardiac rhythm for 12 to 24 hours in the ICU before transferring to the critical care unit [26]. However, ICU admission can be omitted if proper pre- and post-procedure evaluations are completed [28]. Early discharge within 24 to 48 hours can be considered as it has no difference in mortality, stroke, and readmission as compared with late discharge after 48 hours [26]. This approach will also reduce the overall cost and staff workload. The patients are given heparin during the procedure, and a combination of aspirin and clopidogrel for six months post-procedure [29].

Efficacy of TAVI versus SAVR

There are several randomized controlled trials and large registries that studied the efficacy of different valves used in TAVI. Medtronic CoreValve, Edwards SAPIEN, and LOTUS Valve (Boston Scientific) are the available options. The safety of these valves was established by the PARTNER, CoreValve Pivotal, and RESPOND (Rivastigmine to Stabilise Gait in Parkinson's Disease) trials, respectively. Initially, the PARTNER trials discovered the efficacy of the Edwards SAPIEN valve by using two cohort populations. The PARTNER A trial compared the Edwards SAPIEN valve with SAVR in high surgical risk patients, and the PARTNER B cohort compared the Edwards SAPIEN valve with medical therapy in nonsurgical patients. The PARTNER A trial reported one-year mortality of 24.2% in the TAVI group and 26.8% in the SAVR group (p = 0.44). The two-year mortality was 33.9% in the TAVI group and 35% in the SAVR group (p = 0.78). The rate of stroke and transient ischemic attack (TIA) was 8.3% in the TAVI group and 4.3% in the SAVR group (p = 0.04) at one year. The rate of major vascular complications was 11.0% with TAVI and 3.2% with SAVR. The major bleeding episodes were 9.3% in TAVI and 19.5% in SAVR, and atrial fibrillation was reported as 8.6% in TAVI and 16.0% in SAVR. The other cohort, in the PARTNER B trial, reported two-year mortality of 43.4% with TAVI versus 68.0% with medical therapy. The stroke rate at two years was 13.8% with TAVI but only 5.5% with medical therapy [9].

On the other hand, the CoreValve Pivotal trial used the self-expandable transcatheter valve and compared it with medical and surgical treatment. The mortality rate at one year was 14.1% in the TAVI group, whereas it was 18.9% in the SAVR group. The CoreValve Extreme Risk US Pivotal Trial reported a 26% rate of major stroke and all-cause mortality at one year as compared with 43% with a pre-specified estimate with medical therapy [9,30]. Another trial, RESPOND, evaluated outcomes of TAVI with the LOTUS Valve. The rate of mortality and stroke at 30 days was 2.6% and 3.3%, respectively. It was effective in increasing the aortic valve area from 0.7 cm2 at baseline to 1.8cm2 at discharge [31].

**Figure 1:**
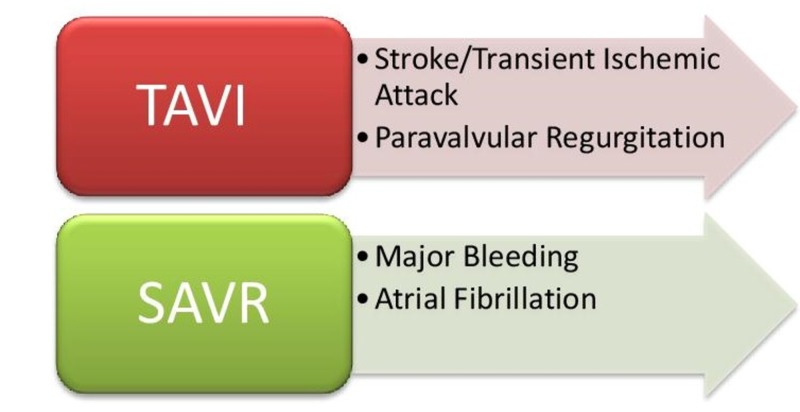
Complications of TAVI and SAVR TAVI, transcatheter aortic valve implantation; SAVR, surgical aortic valve replacement

Hence, with the use of the Edwards SAPIEN, the mortality rate was comparably the same in both the TAVI and SAVR groups. There was an increased risk of stroke, TIA, paravalvular regurgitation, and major vascular complications with TAVI, whereas more cases of major bleeding and atrial fibrillation were reported in the SAVR group. The mortality rate and repeat hospitalization were lower with TAVI as compared with medical management. However, the rate of stroke remained high in the TAVI group. The use of the Medtronic CoreValve showed lower mortality at two years as compared with SAVR. It also demonstrated low rates of all-cause mortality and major stroke as compared with medical therapy. The results with the LOTUS Valve demonstrate they are also safe for implementing in clinical practice. The major adverse effects of the TAVR and SAVR are shown in Figure 1.

Follow-up monitoring

Post-procedural echocardiography should be performed before patient discharge, within 30 days of the procedure, after one year, and annually later on. Other modes of imaging can be used, if necessary. The long-run follow-up is pivotal in monitoring valve function and durability. Along with that, patients should also be clinically monitored for symptoms of congestive cardiac failure, infection, and embolism [32].

Complications

The procedure can have a wide array of peri-procedural and long-term complications. These range from bleeding, annular rupture, stroke, cardiac, and renal ischemia. There is a high chance of valvular malposition/dysfunction. The mechanical prosthesis requires lifelong anticoagulation and has high chances of lifetime bleeding and reoperation. The valvular bioprosthesis can have structural or nonstructural deterioration. Irreversible structural damage is due to tissue degeneration and proliferation, leading to intrinsic changes in the valve, tear, calcification, or pannus formation. Nonstructural valve deterioration is due to paravalvular regurgitation, prosthesis-patient mismatch, malposition, and embolization. Other complications can be thrombosis and endocarditis [32].

Differences in mortality of TAVI versus SAVR

**Figure 2:**
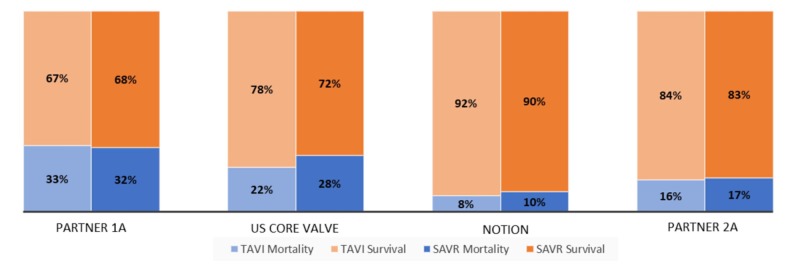
This graph compares the mortality in the TAVI group versus the SAVR group in four different randomized clinical trials. These trials assess the primary outcomes of all-cause mortality at two-year follow-up TAVI, transcatheter aortic valve implantation; SAVR, surgical aortic valve replacement

The graph in Figure 2 compares mortality due to SVAR and TAVI in four different randomized clinical trials. The graph assesses the primary outcome of all-cause mortality at the two-year follow-up.

Future implications

TAVI, with a minimalistic approach, has been increasingly used in many centers. With increasing procedural experience, improvements in patient selection, and newer device performance, the rate of mortality and complications will drop. More modern devices continue to offer promising features such as proven durability, small delivery catheter, better positioning, and retrieval mechanism [31]. These improved traits will markedly shift the conventional practices over time.

Gaps in knowledge

The quest for identifying the optimal method for the management of AS has just started, and there are several gaps in our knowledge that need to be investigated. Studies should investigate the efficacy of elective SAVR in asymptomatic patients with severe AS. The criteria for choice between TAVI and SAVR must be further established in low-operative risk patients. Studies should be conducted to evaluate the factors responsible for conduction disturbances to minimize pacemaker implantations [33]. Furthermore, patients who undergo TAVI should be studied long term (i.e., beyond five years) to identify complications and evaluate the durability of the valves.

## Conclusions

TAVI is a minimally invasive intervention to treat severe symptomatic AS as compared with SAVI. TAVI has better mortality outcomes as compared with SAVR. However, patients who undergo TAVI are at a high risk of major vascular events such as stroke; therefore, a risk-benefit assessment should be performed before going forward with an intervention of TAVI or SAVR for AS.
